# *MDM2* Amplification and *PI3KCA* Mutation in a Case of Sclerosing Rhabdomyosarcoma

**DOI:** 10.1155/2013/520858

**Published:** 2013-05-20

**Authors:** Ken Kikuchi, George R. Wettach, Christopher W. Ryan, Arthur Hung, Jody E. Hooper, Carol Beadling, Andrea Warrick, Christopher L. Corless, Susan B. Olson, Charles Keller, Atiya Mansoor

**Affiliations:** ^1^Pediatric Cancer Biology Program, Department of Pediatrics, Papé Family Pediatric Research Institute, Oregon Health & Science University, 3181 S.W. Sam Jackson Park Road, Mail Code L321, Portland, OR 97239-3098, USA; ^2^Department of Pathology, Oregon Health & Science University, 3181 S.W. Sam Jackson Park Road, Mail Code L41, Portland, OR 97239-3098, USA; ^3^Division of Hematology-Oncology, Department of Medicine, Oregon Health & Science University, Portland, OR 97239, USA; ^4^Department of Radiation Oncology, Oregon Health & Science University, Portland, OR 97239, USA; ^5^Molecular and Medical Genetics, Oregon Health & Science University, Portland, OR 97239, USA

## Abstract

A rare sclerosing variant of rhabdomyosarcoma characterized by prominent hyalinization and pseudovascular pattern has recently been described as a subtype biologically distinct from embryonal, alveolar, and pleomorphic forms. We present cytogenetic and molecular findings as well as experimental studies of an unusual case of sclerosing rhabdomyosarcoma. The primary lesion arose within the plantar subcutaneous tissue of the left foot of an otherwise healthy 23-year-old male who eventually developed pulmonary nodules despite systemic chemotherapy. Two genetic abnormalities identified in surgical and/or autopsy samples of the tumor were introduced into 10T1/2 murine fibroblasts to determine whether these genetic changes cooperatively facilitated transformation and growth. Cytogenetic analysis revealed a complex abnormal hyperdiploid clone, and *MDM2* gene amplification was confirmed by fluorescence in situ hybridization. Cancer gene mutation screening using a combination of multiplexed PCR and mass spectroscopy revealed a *PIK3CA* exon 20 H1047R mutation in the primary tumor, lung metastasis, and liver metastasis. However, this mutation was not cooperative with *MDM2* overexpression in experimental assays for transformation or growth. Nevertheless, *MDM2* and *PIK3CA* are genes worthy of further investigation in patients with sclerosing rhabdomyosarcoma and might be considered in the enrollment of these patients into clinical trials of targeted therapeutics.

## 1. Introduction

Rhabdomyosarcoma (RMS) is subdivided into three major variants: embryonal, alveolar, and pleomorphic. Embryonal and alveolar subtypes are commonest sarcomas of childhood and adolescence. Better clinical outcome is associated with botryoid and spindle cell variants of embryonal RMS. In particular, the spindle cell variant in childhood is considered to be of low malignant potential with excellent overall patient survival. Pleomorphic RMS is rare and highly aggressive adult sarcomas typically arising in the deep soft tissue of the extremities. Even rarer are recently described spindle cell and sclerosing variants of RMS in adults. Due to their rarity, the experience with the newer subsets is limited but appears to show poor outcome in adults. Sclerosing variant of RMS as a distinct entity was initially reported in three cases by Mentzel and Katenkamp in 2000 [1]. Histologically the tumor is characterized by polygonal to spindle-shaped neoplastic cells forming anastomosing cords in pseudovascular clefts and a highly sclerotic, hyalinized matrix. Rare rhabdomyoblasts can be seen and the skeletal muscle differentiation is evidenced by immunoreactivity for desmin, MyoD1, and myogenin. In a subsequent series of four additional cases, Folpe considered these tumors to be either highly unusual variants of adult embryonal rhabdomyosarcoma or an entirely novel subcategory of rhabdomyosarcoma [2]. In these and other reported cases, lesions arose slightly more commonly within the distal extremities, but others have been observed in the head and neck [3], retroperitoneum, and scrotum [4]. There is no particular gender predominance in patients ranging in age from young children to older adults. With fewer than 30 cases reported, genetic analysis has been limited. To date, only six karyotypes [5–7] and one comparative genomic hybridization [8] have been reported showing aneuploidy with numerous chromosomal gains but noregional amplifications [5–7]. Reciprocal translocations typical of alveolar rhabdomyosarcoma, either t(1;13)(p36;q14) or t(2;13)(q35;q14), have not been present. In one case, comparative genomic hybridization revealed loss of chromosome region10q22, loss of chromosome Y, and trisomy of chromosome 18 [8]. Recently, single nucleotide polymorphism genotyping of a sclerosing rhabdomyosarcoma revealed amplification within the 12q13-15 region, including the genes *SAS, GLI*, *CDK4*, and *MDM2* [9]. Herein we describe a case of sclerosing rhabdomyosarcoma studied by karyotyping, mutational screening of 53 cancer genes, and *in vitro* correlative analyses.

## 2. Materials and Methods

Representative 5-*μ*m thick sections of formalin-fixed, paraffin-embedded tissue from the open biopsy specimen were stained with hematoxylin and eosin. For immunohistochemistry, three to four *μ*m unstained paraffin-sections were prepared. Immunohistochemical staining was performed on Ventana (Tucson, AZ, USA) XT stainers. Primary antibodies are as follows: smooth muscle actin (1A4, Dako, Carpinteria, CA, USA), desmin (DE-R-11, Ventana, Tuscon, AZ, USA), myogenin (F5D, Dako), H-Caldesmon (h-CD, Dako), CD99 (H036-1.1, Cell Marque, Roklin, CA, USA), S100 (Polyclonal, Ventana), HMB45 (HMB-45, Dako), cytokeratin cocktail (AE1/AE3/PCK26, Ventana), EMA (E29, Cell Marque), CD34 (QBEnd/10, Ventana), and CD31 (IA10, Cell Marque). Ventana's Ultraview diaminobenzidine chromogen kit was used for secondary antibodies and detection.

### 2.1. Cytogenetic Analysis

Fresh tumor in RPMI tissue culture medium (Invitrogen, Carlsbad, CA, USA) was sent to the Cytogenetics Laboratory within 2 hours from time of biopsy. The sample was enzymatically dissociated using Type I Collagenase (cat. no. LS004196; Worthington Biochemical, Lakewood, NJ, USA) at a final concentration of 1 unit/mL for 90 minutes. The dissociated cells were cultured in RPMI plus 15% fetal bovine serum (Irvine Scientific, Santa Ana, CA, USA) or a combination of this with Chang (Irvine Scientific) complete medium in closed tissue culture flasks for 5 and 7 days. Cell harvest and preparation of slides were performed according to standard laboratory protocol. Twenty-four G-banded (trypsin/Wright stain) metaphase cells were analyzed at approximately the 300 band level.

### 2.2. Fluorescence In Situ Hybridization

Fluorescence in situ hybridization was performed using Abbott probes for *FOXO1A* (13q34) and *MYCN* (2p24.1) with a control probe for the 2 centromere (CEP 2) (Abbott Molecular, Des Plaines, IL, USA) and the ZytoVision *MDM2* (12q14.3-15) probe with a 12 centromeric probe (CEN 12) as control (ZytoVision, Bremerhaven, Germany). Hybridization methods were per manufacturer's instructions and using a HYBritehybridization system (Abbott Molecular, Des Plaines, IL, USA). Interphase cells were evaluated using a Nikon Eclipse E800 (Nikon Corporation, Tokyo, Japan). One hundred interphase cells were scored for the *FOXO1A* and *MYCN*/CEP2 probes. Two hundred interphase cells were scored for the *MDM2*/CEN12 probe set. The cells were captured on a CytoVision computer system with a digital camera (Genetix-Applied Imaging, San Jose, CA, USA).

### 2.3. Mass Spectrometry-Based Mutation Detection

Multiplex mutation analysis was performed on the Mass Array system (Sequenom, San Diego, CA, USA) using a panel that screens for 647 mutations across 53 genes, exactly as previously described [10]. A *PIK3CA* H1047R mutation identified by this approach was confirmed by Sanger sequencing.

### 2.4. Cell Culture

10T1/2 cells and 10T1/2-H1047R cells have been previously described [11]. Cells were cultured in 4.5 g/L glucose DMEM (Invitrogen) supplemented with 10% FBS, 100 U/mL penicillin, and 100 *μ*g/mL streptomycin (Sigma-Aldrich, St. Louis, MI, USA) in 5% CO_2_ in air at 37°C.

### 2.5. Vector Transfection

The* MDM2* vector was kindly provided by Dai et al. (Department of Biochemistry and Molecular Biology, School of Medicine, Oregon Health and Science University) [12]. Transient transfections were carried out using Lipofectamine 2000 (Invitrogen/Life Technologies, Grand Island, NY, USA). pcDNA3 empty vector (Dharmacon-Thermo Fisher Scientific, Waltham, MA, USA) was used as control.

### 2.6. Immunoblotting

10T1/2 cells were lysed in radioimmunoprecipitation assay (RIPA) buffer or NP40 buffer containing both protease and phosphatase inhibitor (Sigma-Aldrich). The lysates were homogenized and centrifuged at 8000 g for 10 minutes. The resulting supernatants were used for immunoblot analysis by mouse anti-*β*-actin (cat. A1978; Sigma), mouse anti-MDM2 (cat. sc-965; Santa CruzBiotechnology, Dallas, TX, USA), rabbit anti-Akt (cat. no. 9272; Cell Signaling, Danvers, MA, USA) or rabbit anti-phospho-Akt (Ser473) (cat. no. 4060; Cell Signaling Technology, Beverly, MA, USA).

### 2.7. Soft-Agar Colony Formation Assay

10T1/2 cells were plated at 6 × 10^3^ cells per well in a 6-well plate with soft-agar with DMEM and 10% fetal bovine serum (SeePlaqueAgarose; cat. 50101, Lonza, Allendale, NJ, USA). *MDM2* vector or empty vector was transfected to the cells before plating the top layer. After the cells were plated 14 days, colonies were counted.

### 2.8. *In Vitro* Growth Assay

10T1/2 cells were plated at 2 × 10^3^ cells of each cohort per well in a 96-well plate. *MDM2* vector or empty vector was transfected to the cells before plating the top layer. After cell incubations, *in vitro* growth was assayed using CellTiter 96 AQueous One Solution Cell Proliferation Assay system (Promega, Madison, WI, USA) and SpectraMax M5 luminometer (Molecular Devices, Sunnyvale, CA, USA).

## 3. Results

### 3.1. Case Report, Histopathological Diagnosis, and Cytogenetics Analysis

**Figure 1:**
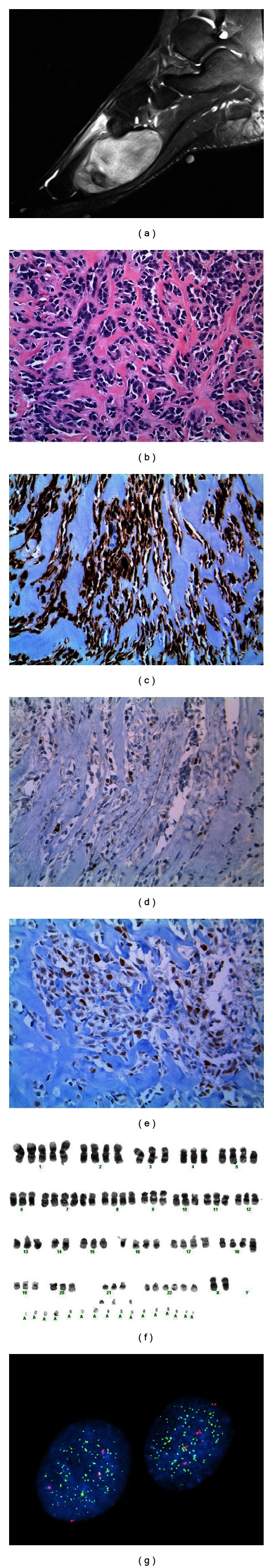
(a) MRI imaging. (b) Diagnostichistology of the tumor demonstrating round hyperchromatic to spindled cells surrounded by a densely hyalinized stroma. Hematoxylin-eosin, original magnification ×200. (c) Desmin immunohistochemical stain showing strong diffuse cytoplasmic expression of desmin. (d) Although sparse, individual cells demonstrated strong nuclear staining with myogenin. Myogenin immunohistochemical stain. (e) Several cells demonstrated strong nuclear staining with MDM2 immunohistochemical stain. (f) Representative G-banded karyogram demonstrating a complex abnormal hyperdiploid clone. These cells contained from 20 to 50 double minutes, shown by fluorescence in situ hybridization (FISH) to be amplification of the *MDM2* gene. (g) Fluorescence in situ hybridization (FISH) with Zytovision *MDM2* probe (green) and a 12 centromere probe as control (red) revealed a subset of interphase cells, as shown here, exhibiting multiple green signals confirming *MDM2 *amplification.

An otherwise healthy 23 year-old man presented with a 6-month history of an enlarging mass within the plantar aspect of his left foot. MRI revealed an 8 cm mass infiltrating the plantar surface, extending through the dorsal surface, and circumferentially involving the 2nd through 4th metatarsals (Figure 1(a)). Open biopsy was performed and light microscopy revealed heavily hyalinized matrix with poorly formed alveolar spaces lined by round hyperchromatic cells (Figure 1(b)). Some cells had scant eosinophilic cytoplasm and other areas showed spindling of tumor cells. Mitotic activity was easily identified. Immunohistochemical staining highlighted skeletal muscle differentiation with strong, diffuse positivity for desmin, and patchy nuclear myogenin positivity (Figures 1(c) and 1(d), resp.). There was scattered nuclear MDM2 immunoreactivity within several cells (Figure 1(e)), whilst a CDK4 immunostain was negative (data not shown). In concert with the marked stromal sclerosis, these results were consistent with the pattern previously reported for sclerosing rhabdomyosarcoma.

Cytogenetic analysis of tumor from the original biopsy revealed fourteen of twenty-four metaphase cells to have a complex hyperdiploid (near-triploid) karyotype. These cells contained approximately 20 to 50 double minutes, shown by FISH (below) to include amplification of the *MDM2* gene. The karyotype, described according to ISCN (2009) [13], was 71~95<3n>,XX,−Y,+1,+add(1)(p?13),+2,+5,+7,+7,+8,+8, 14,+16,+17,+add(18)(q?22),add(19)(q?13.3)×2,+22,+22,+22, +20~50dmin.ish dmin(MDM2×20~50)[cp14]/46,XY[9] (Figure 1(f)).

Interphase fluorescence in situ hybridization (FISH) showed that 15.5% of 200 nuclei had amplification of *MDM2*. These results confirmed amplification of *MDM2 *in the form of the double minutes observed in the metaphase karyotype, as well as the presence of multiple copies of chromosome 12 (Figure 1(g)). FISH results for *MYCN* and for the *FOXO1A* break-apart probe were within normal limits (data not shown).

Staging studies, including operative sampling of left inguinal and iliac lymph nodes due to an abnormal PET scan, did not reveal evidence of metastatic disease. The patient underwent systemic chemotherapy with vincristine, dactinomycin, and cyclophosphamide as a part of a clinical trial. He was offered a below-the-knee amputation, but he declined in favor of radiation. He received a total dose of 50.4 Gy/28 fractions and had an excellent response in the foot. Unfortunately, he developed pulmonary metastases immediately upon the completion of chemotherapy. Subsequent therapies included irinotecan with temozolomide, doxorubicin with ifosfamide, and sorafenib, none of which resulted in clinical response. He also underwent surgical resection of a massive and symptomatic right lower lung metastasis and received several courses of palliative radiation therapy. The patient expired 25 months after diagnosis.

**Figure 2:**
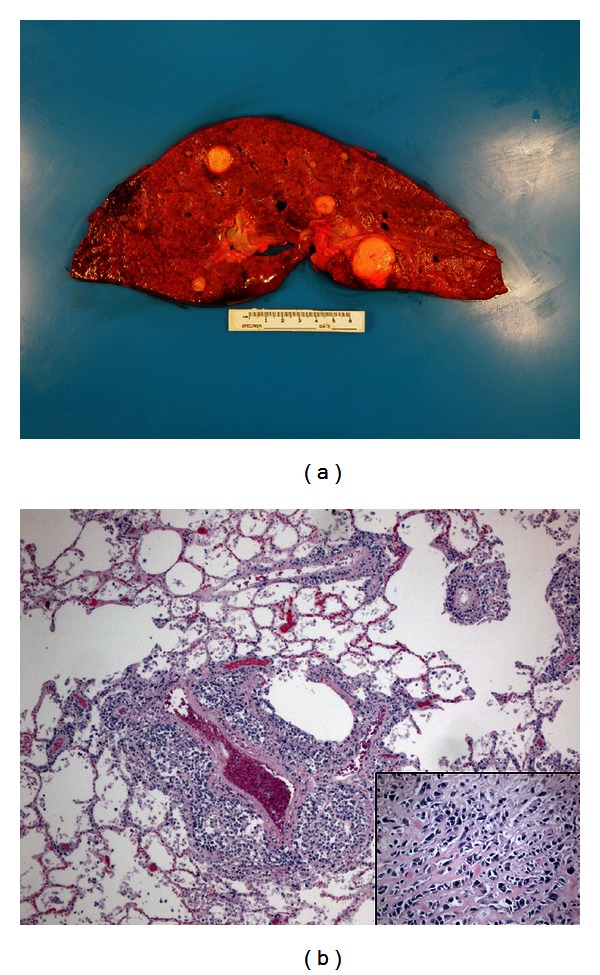
Disseminated disease at necropsy. (a) Cross-section of liver demonstrating metastatic nodules. (b) Histological section of lung with metastatic sclerosing rhabdomyosarcoma. Hematoxylin-eosin, insert original magnifications ×200.

A complete autopsy was performed twenty seven hours after death. Tumor masses were present in all lung lobes, nearly replacing the right lung. There were multiple metastases in both liver lobes, adjacent to esophagus, in the right adrenal, and in the right frontal cortex of the brain, as well as invading the chest wall, diaphragm, and multiple vertebrae. Fresh tissue was obtained sterilely for culture and frozen aliquots from the right chest, left upper lobe, and liver as well as normal skin and skeletal muscle (frozen only) immediately after opening of the body. Histologically the tumor was similar to that in the prior tumor biopsy with somewhat more cytologicatypia and a range of 30 to 90% necrosis (Figure 2).

Genotyping performed on tumor from the autopsy revealed a *PIK3CA* exon 20 H1047R (catalytic domain) mutation in the primary tumor, lung metastasis, and liver metastasis. No other mutations were identified in the genes listed in Table 2.

### 3.2. *In Vitro* Growth Analysis of Combined *MDM2* Amplification and *PIK3CA*-Activated Mutation

**Figure 3:**
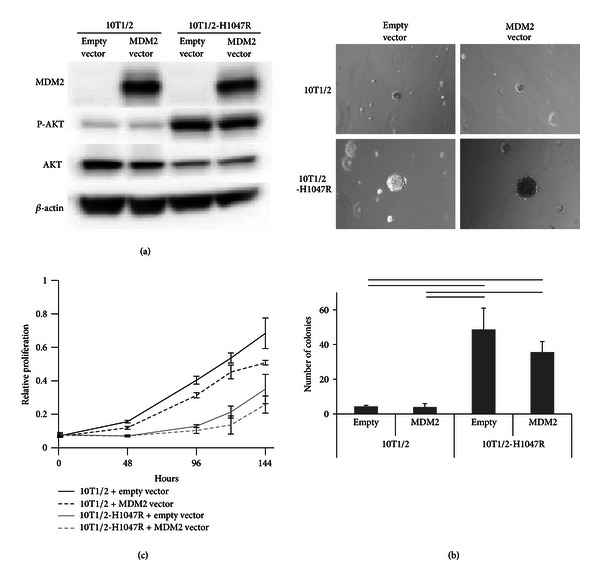
(a) Western blotting of MDM2, Akt, phosphoAkt, and *β*-actin. Total cell lysates were isolated 48 h after transfection. (b) Soft-agar colony formation assay (means ± SD, *n* = 3). Black line shows significant difference (*P* < 0.05). (c) Relative proliferation rates of 10T1/2 cells and 10T1/2-H1047R cells transfected with MDM2 vector or empty vector, as measured by MTS assay (means ± SD, *n* = 3).

To investigate the effect of combined *MDM2* amplification and the *PIK3CA* activating mutation, 10T1/2-PIK3CA H1047R fibroblasts transfected with an *MDM2* overexpression construct versus empty vector control were studied. As expected, the *MDM2* vector induced MDM2 protein overexpression (Figure 3(a)). Similarly, 10T1/2 cells harboring the *PIK3CA* H1047R mutation showed higher Akt phosphorylation compared with control 10T1/2 cell (Figure 3(a)). Next, we compared anchor-independent growth and anchorage-dependent proliferation of 10T1/2 control cells and 10T1/2-H1047R cells with or without the *MDM2* vector. The *PI3KCA* H1047R mutation induced anchor-independent growth, but *MDM2* transient transfection could not alone induce anchor-independent growth or accelerated proliferation in control 10T1/2 cells (Figures 3(b) and 3(c)). These results suggest lack of synergy between *MDM2* amplification and *PI3KCA* mutation with respect to the tumor cell-autonomous growth characteristics tested.

## 4. Discussion

In contrast to childhood RMS, the adult variants are believed to be highly aggressive irrespective of the morphologic subtype. The pleomorphic variant of RMS, exclusively seen in adults, has been extensively studied. Recently described variants in adults include spindle cell and sclerosing RMS. One of the limitations of understanding biology of these new entities is the overall rarity of such cases. It has also been noted that about 15–20% cases of adult spindle cell RMS show morphological overlap with the sclerosing variant [14, 15]. This observation suggests close relationship between the two subsets and raises the possibility of morphological variation of a single-tumor type. Future molecular and genetic studies will help address this issue, but for now these two variants are co-classified as a new distinct subset of adult RMSs [16].

The sclerosing variant of rhabdomyosarcoma presents a significant diagnostic challenge. From its hyalinizing pseudovascular appearance and sometimes focal and dot-like desmin and myogenin expression, these tumors could easily be mistaken for variants of angiosarcoma, extraskeletal myxoid chondrosarcoma, osteosarcoma, or sclerosing epithelioid fibrosarcoma.

Sclerosing rhabdomyosarcoma is an aggressive sarcoma. Among the original three cases reported by Mentzel and Katenkamp, one exhibited progressive pulmonary metastases despite systemic chemotherapy [1]. In one of the four cases reviewed by Folpe et al., there were extensive intra-abdominal and lymph node metastases, and this patient expired within 5 years of diagnosis [2]. None of the three pediatric cases described by Zambrano et al. had metastases [7]. However, in a pediatric case of deltoid tumor recently evaluated by Bouron-Dal Soglio et al., both pulmonary and axillary nodal metastases were present [9].

Among the few karyotypes reported, no characteristic numerical alteration of the chromosomal complement has emerged (Table 1). None of the cases have shown reciprocal translocation t(1;13)(p36;q14) or t(2;13)(q35;q14), which are characteristic of alveolar rhabdomyosarcoma.

Bouron-Dal Soglio et al. reported a highly complex aneuploid pattern by SNP array, including whole chromosome trisomy 5, 7, 8, 11, 15, 16, 20, and 22; tetrasomy 4 and 18; and monosomy 1, 2, 3, 9, 10, 13, 14 [9]. Their analysis revealed amplification at region 12q13-15 which included the *HMGA2 *and *MDM2 *genes but not *CDK4/SAS/GL1*. Both the initial and recurrent lesions possessed identical amplifications. Their pattern was similar to an analysis of 38 well-differentiated/dedifferentiated liposarcomas which revealed overexpression of both *HMGA2 *and *MDM2 *in all cases, but with an inconsistent amplification of *CDK4* in 13% of cases [17]. Amplification and overexpression of *MDM2, CDK4, GL1 *and *SAS *genes of the 12q13-15 region has been associated with other sarcomas such as well-differentiated and dedifferentiated liposarcoma, leiomyosarcoma, and a significant subset of alveolar and embryonal rhabdomyosarcomas [18] and even sclerosing epithelioid fibrosarcoma [19].

Ours is the second reported case of sclerosing rhabdomyosarcoma in which *MDM2 *amplification has been demonstrated [9]. The histological sections did not show areas resembling spindle cell RMS, and hence was a pure variant. Despite some clinical similarities to alveolar RMS, including age range and frequent location within the extremities, translocations involving the *FOXO1A *(also referred to as *FOXOA10*) gene have been absent. Our case further confirms sclerosing RMS as a distinct diagnostic entity and implicates *MDM2 *amplification in the pathogenesis of this tumor. This may aid in the diagnosis of future cases. Aside from the potential diagnostic significance of this finding, enhanced *MDM2 *expression or related p53 abnormalities, if present, may render sclerosing RMS vulnerable to an ever-expanding array of small molecule inhibitors. For example, nutlin specifically blocks p53-MDM2 binding, thus restoring the tumor suppressive activity of p53 [20]. Other potential clinical agents may have relevance: RO5044537 (RG7112) is a member of the nutlin family and is the first MDM2 antagonist to be assessed clinically [21]. APR-246, the analog of PRIMA-1, restores wild-type conformation to mutant p53 by binding to the core and induces apoptosis in human tumor cells [22]. Moreover, arsenic trioxide can indirectly activate p53 via Wip1 phosphatase inhibition [23].

Somatic mutations of *PIK3CA* have been reported in many cancer types, including liver (36%), breast (8–40%), colon (14–32%), and ovarian cancer (6–12%) [24]. Barretina et al. first described *PIK3CA* mutation in soft-tissue sarcomas uncovered by large-scale mutational analysis of the entire coding regions of all the major classes of PI3K [25]. Recently, *PIK3CA* mutations were found in 5% of embryonal RMS [26], but these mutations have not been reported in sclerosing RMS. Functionally, the H1047R mutation in the catalytic domain of p110*α* has transforming activity via Akt phosphorylation [27]. Akt overphosphorylation has been observed in 42-43% of alveolar RMS and 35–55% of embryonal RMS [28]. These results suggest that the PIK3CA-Akt pathway may be important for RMS and an attractive therapeutic target for cancer intervention in RMS. Downstream mediators are potentially druggable with AKT inhibitors such as MK2206 [29] or mTOR inhibitors like rapamycin, whereas dual PI3K/mTOR inhibitor such as PI-103 may be of special benefit. Interestingly, PI-103 has been reported to act synergistically with Nutlin-3 to induce apoptosis in a wild-type p53-dependent fashion [30].

To investigate the effect of combination with *MDM2* amplification and *PIK3CA*-activated mutation in proliferation *in vitro*, we performed soft-agar assay and proliferation assay using 10T1/2-*PIK3CA* H1047R cells transfected with an *MDM2* overexpression construct versus empty vector control. In this fibroblast-based model we could not identify a growth advantage of combined *MDM2* amplification and *PIK3CA*-activated mutation. Other models may be necessary to determine whether *MDM2 *amplification and *PIK3CA* H1047R mutation facilitate metastasis or tumorigenesis [31]. And other molecular changes may play a role in sclerosing RMS.

Given the discussion above that *MDM2* and *PIK3CA* can be targeted by emerging small molecule inhibitors, mutations in these loci may warrant further investigation in larger cohorts of patients with sclerosing RMS. However, in a tumor as rare as sclerosing RMS, personalized science and accompanying tailored treatment approaches may be needed.

## Figures and Tables

**Table 1:** Summary of reported cytogenetic abnormalities in sclerosing rhabdomyosarcoma.

Author	Year	Cases	Notes
Mentzel and Katenkamp [1]	2000	3	No ARMS fusion transcripts due to t(1;13) or t(2;13) found by RT-PCR
Folpe et al. [2]	2002	4	Cases 1 and 3: inadequate RNA for RT-PCR. Case 2: no evidence of *PAX3/FOXO1A *or *PAX7/FOXO1A *fusion gene by RT-PCR. Case 4: not tested due to the absence of nonembolized tissue
Vadgama et al. [4]	2004	1	No ARMS fusion transcripts by RT-PCR
Chiles et al. [5]	2004	13	4 cases negative for ARMS fusion transcripts (*PAX3- *and *PAX7-FOXO1A*) by RT-PCR
Croes et al. [6]	2005	1	Tumor cells negative for *FOXO1A*-disrupting translocation by FISH; karyotype: 44–49,XX,+del(1)(p22)[2],+11,+16[5],+18[12],+21[3],−22[cp13]
Zambrano et al. [7]	2006	3	Case 1: normal. Case 2: complex structural and numerical abnormalities with numerous double minutes in most cells; case 3: less complex, with balanced translocation 46,XX,t(5;20)(q31;pl3)
Kuhnen et al. [8]	2006	1	CGH: loss of 10q22, loss of chromosome Y, gain of chromosome 18 (trisomy)
Bouron-Dal Soglio et al. [9]	2009	1	SNP array: complex aneuploid pattern including gains and losses of whole chromosome and an amplification of 12q13-15. FISH analysis showed amplification of *HMGA2 *and *MDM2 *

**Table 2:** Genes screened by solid tumor sequenom panel.

*AKT1 *	*CTNNB1 *	*FGFR1 *	*GNAS *	*KRAS *	*NRAS *	*PIK3R5 *	*STAT1 *
*AKT2 *	*EGFR *	*FGFR2 *	*HRAS *	*MAP2K1 *	*NTRK1 *	*PKHD1 *	*TEC *
*AKT3 *	*ERBB2* (*HER2*)	*FGFR3 *	*IDH1 *	*MAP2K2 *	*NTRK2 *	*PRKCB1 *	*TP53 *
*ALK *	*ERCC6 *	*FGFR4 *	*IDH2 *	*MAP2K7 *	*NTRK3 *	*RAF1 *	
*BRAF *	*FBX4 *	*FOXL2 *	*IGF1R *	*MET *	*PDGFRA *	*RET *	
*CDK4 *	*FBXW7 *	*GNA11 *	*KDR *	*MYC *	*PIK3R1 *	*SMO *	
*CSF1R *	*FES *	*GNAQ *	*KIT *	*NEK9 *	*PIK3R4 *	*SOS1 *	
